# Supplementary material for: Association of adenotonsillectomy with asthma and upper respiratory infection: A nationwide cohort study
Source: PLoS One. 2020 Jul 30;15(7):e0236806. doi: 10.1371/journal.pone.0236806 (PMC7392329; doi:10.1371/journal.pone.0236806)
Supplement: S1 File — (DOCX) [file pone.0236806.s001.docx]

**Online Repository Material**

**METHODS**

***Korea National Health Insurance Service***

The national health insurance service has provided health coverage to the entire South Korean population since 1989. An insured individual pays a national health insurance levy, which is proportional to the individual’s income. Although a user charge exists, it is mandatory for all Koreans to join the Korean NHIS. Each South Korean resident is assigned a unique identification number at birth, which is used by the KNHIS to identify each individual. Thus, healthcare claims data are not omitted or duplicated. The majority (97.1%) of the Korean population are mandatory subscribers. The remaining 3% of the population with low income are covered by the Medical Aid program. Since 2006, data of the Medical Aid beneficiaries have been incorporated into the NHIS database. Therefore, the data extracted from the NHIS database are based on the entire Korean population, eliminating any selection bias. In addition, the KNHIS controls all medical costs among beneficiaries, medical providers, and the government. Almost all medical data, including diagnostic codes, procedures, prescription drugs, and personal information, are included in the KNHIS database. In the KNHIS, the Korean Classification of Diseases, which is a very similar to the International Classification of Diseases, is used as a system of diagnostic practice codes. The present study used KNHIS-NSC data collected from 2002 to 2013, which comprised information from 1,025,340 nationally representative random subjects, accounting for approximately 2.2% of the South Korean population in 2002. Stratified random sampling was performed using 1,476 strata with respect to age (18 groups), sex (two groups), and income level (41 groups: 40 health insurance and one medical aid beneficiary) among the South Korean population of 46 million in 2002. This study used the KNHIS-NSC data (NHIS-2018-2-143) generated by the NHIS.

***Interactive Visualization of Healthcare Data***

We created the interactive visualizations for the number of URI visits based on the equivalence test using Tableau Software; these data may be publicly shared on the Tableau public sites, as follows:

https://public.tableau.com/profile/sweetino#!/vizhome/95CIofdifference0_5inURI/URI.

l
