# Supplementary material for: Association of adenotonsillectomy with asthma and upper respiratory infection: A nationwide cohort study
Source: PLoS One. 2020 Jul 30;15(7):e0236806. doi: 10.1371/journal.pone.0236806 (PMC7392329; doi:10.1371/journal.pone.0236806)
Supplement: S1 Table — (DOCX) [file pone.0236806.s002.docx]

**S1 Table.** Equivalence tests for upper respiratory infections in the postoperative period in patients under 4 years

| **Variable** | **Comparison**  **(mean ± SD)** | **Adenotonsillectomy (mean ± SD)** | **95% CI of the difference (0.5)** | **P value** |
| --- | --- | --- | --- | --- |
| Pre-op visit | 6.6 ± 5.9 | 6.8 ± 6.2 | -0.98 to 1.32 | 0.773 |
| Post-op 1 y visit | 4.6 ± 2.9 | 4.4 ± 2.8 | -0.72 to 0.34 | 0.486 |
| Post-op 2 y visit | 3.7 ± 3.0 | 3.4 ± 2.7 | -0.86 to 0.19 | 0.208 |
| Post-op 3 y visit | 3.2 ± 2.8 | 2.8 ± 2.3 | -0.90 to 0.00 | 0.048 |
| Post-op 4 y visit | 2.9 ± 2.8 | 2.2 ± 2.2 | -1.11 to -0.23 | 0.003 |
| Post-op 5 y visit | 2.8 ± 2.6 | 2.4 ± 2.1 | -0.81 to 0.01 | 0.056 |
| Post-op 6 y visit | 2.6 ± 2.4 | 1.8 ± 1.8 | -1.15 to -0.43 | 0.000 |
| Post-op 7 y visit | 2.3 ± 2.2 | 2.1 ± 2.1 | -0.64 to 0.18 | 0.265 |
| Post-op 8 y visit | 2.0 ± 2.2 | 2.0 ± 2.4 | -0.47 to 0.41 | 0.894 |
| Post-op 9 y visit | 1.7 ± 2.1 | 1.6 ± 1.8 | -0.46 to 0.23 | 0.518 |
| Post-op 10 y visit | 1.0 ± 1.7 | 0.8 ± 1.4 | -0.42 to 0.15 | 0.353 |
| Post-op 11 y visit | 0.4 ± 1.2 | 0.3 ± 0.9 | -0.29 to 0.06 | 0.199 |

Op: operation, SD: Standard deviation, Difference: adenotonsillectomy group - comparison group, CI: Confidence interval
