# Supplementary material for: Association of adenotonsillectomy with asthma and upper respiratory infection: A nationwide cohort study
Source: PLoS One. 2020 Jul 30;15(7):e0236806. doi: 10.1371/journal.pone.0236806 (PMC7392329; doi:10.1371/journal.pone.0236806)
Supplement: S2 Table — (DOCX) [file pone.0236806.s003.docx]

**S2 Table.** Equivalence tests for upper respiratory infections in the postoperative period in patients aged 5-9 years

| **Variable** | **Comparison**  **(mean ± SD)** | **Adenotonsillectomy (mean ± SD)** | **95% CI of the difference (0.5)** | **P value** |
| --- | --- | --- | --- | --- |
| Pre-op visit | 5.3 ± 5.4 | 5.3 ± 5.3 | -0.49 to 0.41 | 0.863 |
| Post-op 1 y visit | 2.7 ± 2.4 | 3.0 ± 2.5 | 0.09 to 0.51 | 0.004 |
| Post-op 2 y visit | 2.3 ± 2.2 | 2.6 ± 2.3 | 0.14 to 0.53 | 0.001 |
| Post-op 3 y visit | 1.9 ± 2.0 | 2.2 ± 2.3 | 0.10 to 0.47 | 0.003 |
| Post-op 4 y visit | 1.7 ± 2.0 | 2.0 ± 2.2 | 0.08 to 0.45 | 0.005 |
| Post-op 5 y visit | 1.7 ± 2.0 | 2.1 ± 2.3 | 0.24 to 0.62 | 0.000 |
| Post-op 6 y visit | 1.6 ± 2.0 | 1.9 ± 2.1 | 0.09 to 0.44 | 0.003 |
| Post-op 7 y visit | 1.4 ± 1.8 | 1.8 ± 2.0 | 0.21 to 0.54 | 0.000 |
| Post-op 8 y visit | 1.3 ± 1.7 | 1.6 ± 1.9 | 0.13 to 0.44 | 0.000 |
| Post-op 9 y visit | 1.2 ± 1.7 | 1.4 ± 1.9 | 0.02 to 0.33 | 0.024 |
| Post-op 10 y visit | 0.7 ± 1.3 | 0.9 ± 1.6 | 0.06 to 0.32 | 0.004 |
| Post-op 11 y visit | 0.3 ± 0.9 | 0.4 ± 1.2 | 0.01 to 0.20 | 0.027 |

Op: operation, SD: Standard deviation, Difference: adenotonsillectomy group - comparison group, CI: Confidence interval
