# Supplementary material for: Association of adenotonsillectomy with asthma and upper respiratory infection: A nationwide cohort study
Source: PLoS One. 2020 Jul 30;15(7):e0236806. doi: 10.1371/journal.pone.0236806 (PMC7392329; doi:10.1371/journal.pone.0236806)
Supplement: S3 Table — (DOCX) [file pone.0236806.s004.docx]

**S3 Table.** Equivalence tests for upper respiratory infections in the postoperative period in patients aged 10-14 years

| **Variable** | **Comparison**  **(mean ± SD)** | **Adenotonsillectomy (mean ± SD)** | **95% CI of the difference (0.5)** | **P value** |
| --- | --- | --- | --- | --- |
| Pre-op visit | 3.1 ± 3.4 | 3.2 ± 3.6 | -0.45 to 0.59 | 0.801 |
| Post-op 1 y visit | 1.4 ± 1.7 | 1.6 ± 1.9 | -0.05 to 0.49 | 0.110 |
| Post-op 2 y visit | 1.3 ± 1.7 | 1.4 ± 1.8 | -0.15 to 0.38 | 0.402 |
| Post-op 3 y visit | 1.2 ± 1.6 | 1.2 ± 1.6 | -0.20 to 0.27 | 0.762 |
| Post-op 4 y visit | 1.1 ± 1.5 | 1.2 ± 1.6 | -0.17 to 0.30 | 0.570 |
| Post-op 5 y visit | 1.2 ± 1.7 | 1.2 ± 1.6 | -0.31 to 0.17 | 0.562 |
| Post-op 6 y visit | 1.1 ± 1.7 | 1.3 ± 1.7 | -0.08 to 0.43 | 0.178 |
| Post-op 7 y visit | 1.0 ± 1.6 | 1.0 ± 1.5 | -0.22 to 0.22 | 0.992 |
| Post-op 8 y visit | 0.8 ± 1.3 | 0.7 ± 1.2 | -0.27 to 0.09 | 0.340 |
| Post-op 9 y visit | 0.8 ± 1.3 | 0.8 ± 1.2 | -0.17 to 0.19 | 0.925 |
| Post-op 10 y visit | 0.5 ± 1.1 | 0.4 ± 1.0 | -0.21 to 0.08 | 0.371 |
| Post-op 11 y visit | 0.2 ± 0.7 | 0.1 ± 0.5 | -0.16 to 0.01 | 0.064 |

Op: operation, SD: Standard deviation, Difference: adenotonsillectomy group - comparison group, CI: Confidence interval
