# Supplementary material for: Association of adenotonsillectomy with asthma and upper respiratory infection: A nationwide cohort study
Source: PLoS One. 2020 Jul 30;15(7):e0236806. doi: 10.1371/journal.pone.0236806 (PMC7392329; doi:10.1371/journal.pone.0236806)
Supplement: S4 Table — (DOCX) [file pone.0236806.s005.docx]

**S4 Table.** Equivalence tests for upper respiratory infections in the postoperative period in patients living in Seoul

| **Variable** | **Comparison**  **(mean ± SD)** | **Adenotonsillectomy (mean ± SD)** | **95% CI of the difference (0.5)** | **P value** |
| --- | --- | --- | --- | --- |
| Pre-op visit | 4.8 ± 4.9 | 4.8 ± 5.0 | -0.66 to 0.78 | 0.870 |
| Post-op 1 y visit | 2.7 ± 2.6 | 3.0 ± 2.4 | -0.04 to 0.67 | 0.078 |
| Post-op 2 y visit | 2.3 ± 2.4 | 2.8 ± 2.5 | 0.15 to 0.87 | 0.005 |
| Post-op 3 y visit | 2.0 ± 2.2 | 2.1 ± 2.4 | -0.18 to 0.51 | 0.354 |
| Post-op 4 y visit | 1.8 ± 2.2 | 1.8 ± 2.1 | -0.23 to 0.39 | 0.611 |
| Post-op 5 y visit | 1.7 ± 2.2 | 1.9 ± 2.2 | -0.21 to 0.44 | 0.470 |
| Post-op 6 y visit | 1.7 ± 2.1 | 1.6 ± 1.7 | -0.32 to 0.21 | 0.687 |
| Post-op 7 y visit | 1.6 ± 2.0 | 1.6 ± 1.9 | -0.23 to 0.33 | 0.739 |
| Post-op 8 y visit | 1.4 ± 1.8 | 1.4 ± 1.7 | -0.21 to 0.30 | 0.737 |
| Post-op 9 y visit | 1.3 ± 1.7 | 1.2 ± 1.7 | -0.35 to 0.14 | 0.407 |
| Post-op 10 y visit | 0.7 ± 1.3 | 0.7 ± 1.5 | -0.14 to 0.29 | 0.481 |
| Post-op 11 y visit | 0.3 ± 0.9 | 0.3 ± 0.8 | -0.14 to 0.10 | 0.705 |

Op: operation, SD: Standard deviation, Difference: adenotonsillectomy group - comparison group, CI: Confidence interval
