# Supplementary material for: Association of adenotonsillectomy with asthma and upper respiratory infection: A nationwide cohort study
Source: PLoS One. 2020 Jul 30;15(7):e0236806. doi: 10.1371/journal.pone.0236806 (PMC7392329; doi:10.1371/journal.pone.0236806)
Supplement: S5 Table — (DOCX) [file pone.0236806.s006.docx]

**S5 Table.** Equivalence tests for upper respiratory infections in the postoperative period in patients living in other areas

| **Variable** | **Comparison**  **(mean ± SD)** | **Adenotonsillectomy (mean ± SD)** | **95% CI of the difference (0.5)** | **P value** |
| --- | --- | --- | --- | --- |
| Pre-op visit | 5.1 ± 5.3 | 5.1 ± 5.3 | -0.41 to 0.41 | 0.987 |
| Post-op 1 y visit | 2.7 ± 2.5 | 2.9 ± 2.6 | -0.01 to 0.39 | 0.056 |
| Post-op 2 y visit | 2.3 ± 2.3 | 2.4 ± 2.3 | -0.07 to 0.29 | 0.224 |
| Post-op 3 y visit | 1.9 ± 2.1 | 2.1 ± 2.1 | -0.04 to 0.29 | 0.134 |
| Post-op 4 y visit | 1.8 ± 2.1 | 1.9 ± 2.1 | -0.06 to 0.27 | 0.218 |
| Post-op 5 y visit | 1.7 ± 2.1 | 2.0 ± 2.1 | 0.08 to 0.40 | 0.004 |
| Post-op 6 y visit | 1.7 ± 2.0 | 1.8 ± 2.1 | -0.01 to 0.31 | 0.067 |
| Post-op 7 y visit | 1.4 ± 1.8 | 1.7 ± 2.0 | 0.11 to 0.41 | 0.001 |
| Post-op 8 y visit | 1.3 ± 1.7 | 1.5 ± 1.9 | 0.05 to 0.34 | 0.009 |
| Post-op 9 y visit | 1.2 ± 1.7 | 1.3 ± 1.8 | 0.03 to 0.30 | 0.020 |
| Post-op 10 y visit | 0.7 ± 1.4 | 0.8 ± 1.5 | -0.01 to 0.21 | 0.086 |
| Post-op 11 y visit | 0.3 ± 0.9 | 0.3 ± 1.1 | -0.03 to 0.14 | 0.000 |

Op: operation, SD: Standard deviation, Difference: adenotonsillectomy group - comparison group, CI: Confidence interval
